# Manuel De Medicine Operatoire, Fonde Sur L’Anatomie Normale Et L’Anatomie Pathologique

**Published:** 1841-05

**Authors:** 


ART. II.—*Manuel de Medicine Operatoire, fonde sur L'Anatomie Normale et L'Anatomie Pathologique. Par* J. F. MALGAIGNE, M. D.

Manual of Operative Surgery, founded on Healthy and Morbid Anatomy. By J. F. MALGAIGNE, M. D.; 18mo., pp. 773. Paris: 1837.

This is one of the numerous manuals which have been issued by the Parisian press, within the last twelve years, on the different departments of medical science; and judging from an attentive examination of its contents, we are disposed to believe that it is quite equal, if not superior, to any of those that have preceded it. M. Malgaigne is well known in France, not only as an able writer, but as an excellent teacher, being attached to the Parisian Faculty of Medicine as *under-professor* of surgical anatomy and operative medicine. The first edition of the present work was published about seven years ago, and although it was by no means small, scarcely two years had elapsed before it was completely exhausted. Soon after its appearance in Paris, it was reprinted in Belgium and translated into the German language; flattering proofs of the estimate placed upon it by the physicians of other countries. In addition to his manual of operative medicine, he is the author of an able dissertation on uterine polypes, of a memoir on a new theory of the human voice, of an essay on the best method of treating lachrymal fistules, of Clinical Souvenirs of the St. Louis Hospital, and finally, of a treatise on surgical anatomy. The latter work, which was published a few years ago, will be noticed more fully in a future number of the journal.

In this edition the author has carefully corrected the errors of the first, and, besides a great many facts and observations introduced into the text, several new articles are added to the work. Indeed, the manner in which he has executed his task is highly creditable to his talents; and his manual may be safely referred to as presenting an accurate outline of the existing state of the science of which it treats. The subject is not, of course, so elaborately discussed as in the celebrated work of Professor Velpeau, because M. Malgaigne has written more particularly for the use of students and the younger members of the profession, and has therefore confined himself within much narrower limits; he has furnished rather a miniature than a grand picture, in which all the features are fully delineated, or set forth in their just proportions. In the composition of his manual he has availed himself freely of the various and accurate information embodied in the writings of Sabatier, Lisfranc, Mayor, Ducamp, Amussat, Percy, Dupuytren, Senn, Velpeau, and others, besides the most important periodical literature of the day. Every operative procedure, no matter by whom devised, improved or modified, is carefully described, and in every instance, at least so far as we are enabled to judge, referred to its appropriate author. In this respect, ample justice has been done to the surgeons of every nation, and, as might be expected, those of the United States are by no means over-looked or neglected. The name of Dr. Mott, known in every portion of the civilized world, not only figures in the preface, but occurs in connexion with some of the boldest operations that have ever been performed on the human subject; that of Physick is also duly noticed; and so are several others of minor note.

As the work of M. Malgaigne is strictly elementary in its character, it would be impossible to present any thing like an analysis of its contents; we shall, therefore, conclude our notice of it with a translation of his remarks upon hydrocele, which have been selected at random, to afford the reader some idea of the manner in which his task has been executed.

"Hydrocele may be divided into several species, namely: first, into *hydrocele by infiltration*, which is nothing else than œdema of the scrotum, and which demands no particular operation for its removal; secondly, into *encysted hydrocele*, which consists in the development most frequently of one or more cysts along the course of the spermatic cord, and which requires the same treatment as similar diseases in other parts of the body; thirdly and lastly, *vaginal hydrocele*, which is seated in the vaginal tunic, and presents itself in several forms. Thus, the tumor may communicate with the peritonæal cavity, as happens when the canal originally existing between these two sacs is not obliterated: in this case the disease forms what is termed *congenital hydrocele.* Again, the vaginal tunic, instead of presenting its ordinary character, may be thickened, lardaceous, cartilaginous, or even osseous; the testicle itself may be altered, and so constitute hydro-sarcocele; in short, the malady may be complicated with varicocele, hernia, and other affections.

1.—*Hydrocele in the Adult.*

"Besides local applications and blisters to the scrotum, to promote the absorption of the effused fluid, numerous operative procedures have been recommended for the cure of this disease, which may be thus stated: puncture, incision, excision, cauterization of the sac, the introduction of the seton or other foreign bodies, and lastly, injection.

I. *Puncture.*—This operation may be performed either with a small trocar or a lancet, the former being preferable.

The integuments should be previously put on the stretch and drawn away from the testicle. The patient sitting on a chair or lying upon the edge of the bed, the tumor is grasped with the hand, so as to make the anterior and inferior part of it project between the thumb and forefinger, while the upper and back part, corresponding to the cord and testicle, is concealed in the palm of the hand. The trocar is then to be plunged from below upwards through the antero-inferior part of the tumor, if we adopt the method of Sabatier, or upwards and slightly backwards, if we follow that of Boyer; the perforator being now withdrawn, the canula is pushed on until we have reason to believe that it will not escape from the vaginal tunic during the evacuation of the fluid. If this precaution be neglected, the fluid might be extravasated into the cellular tissue of the scrotum.

II. *Incision.*—The patient being placed upon his back, the surgeon, standing on the right side, stretches the integuments with the left hand, and with a convex bistoury makes an incision from above downwards in the direction of the longest diameter of the tumor. Arrived at the sac, he makes a slight opening into it, which is afterwards to be enlarged in the line of the external cut, by carrying the instrument upon the index-finger or upon a grooved director. The operation being completed, the wound is filled with lint, which is to be kept in until suppuration is established. As a modification of this method we may mention that of Franco, which consists in an incision of about three inches in length, and the introduction of a *tent* of lint, charpie or sponge, steeped in rose oil. Its object, as in the preceding case, is to produce suppuration in the sac.

III. *Excision.*—Of this there are not less than four methods. In that of Douglas, the patient lying on his back as in the operation by incision, the skin of the scrotum is to be divided in such a manner as to form an oval flap, the great diameter of which extends from above downwards. This flap being dissected up and held out of the way, the sac is laid open in its whole length, after which it is dilated with the skin, and removed as far as the base of the spermatic cord. The edges of the wounds are next to be approximated, taking care, nevertheless, to interpose some charpie to promote suppuration.

The operation as performed by Boyer consists in making a superficial incision through the whole length of the anterior and middle part of the tumor, and in separating the skin from the vaginal tunic, the dissection being prolonged nearly to the point of reflection of the membrane over the testicle. Care should be taken not to cut away the cellular substance too closely to the skin, otherwise gangrene might ensue, and to avoid opening the sac before it is completely exposed. The separation being effected, the vaginal tunic is laid open through its whole length, and excised with a pair of scissors as near as possible to the testicle.

The third method is that of Dupuytren, who divides the skin like Boyer, or excises it like Douglas, according as it is necessary or unnecessary to raise a flap of integuments; the edges of the incision are then drawn back, by compressing them with the thumb and forefinger of the left hand, enucleating thus the testicle and vaginal sac. The operation is completed by opening the cyst and cutting it off with a pair of scissors.

The method of Kinderwood, the fourth and last that we shall mention, differs so much from the preceding that it may be regarded as a new operation. Having made a large puncture in the tumor with an abscess lancet, a pair of dissecting forceps is to be introduced into the aperture, with which a small portion of the sac is seized, and snipped off with a bistoury or pair of scissors.

IV. *Cauterization.*—This is performed by applying upon the anterior and lower part of the tumor a bit of caustic potassa large enough to produce an eschar of the size of a franc piece. As soon as there is reason to believe that its action has extended to the vaginal tunic, it is to be carefully removed, and the part dressed with some simple unguent. In eight or ten days the slough separates; in from five to six weeks the vaginal tunic is said to exfoliate, and the cure is finally completed by the manner in which the membrane adheres to the testicle.

V. *The Seton and other Foreign Bodies.*—Pott's method. He made at first a puncture in the ordinary manner at the most dependent part of the tumor, with a trocar three lines in diameter. Through the canula of this instrument he next introduced a silver tube five inches long, to the upper part of the vaginal tunic, so as to be felt there; a probe, six inches and a half in length, having at one end a fine steel trocar, and at the other an eye, armed with a seton, was then conveyed through the latter canula, and brought out at the place already specified. This method seems to comprise the germs of M. Belmas' instruments for his operation for inguinal hernia.

Monro used to leave the trocar-canula in the part; others, however, substitute a piece of bougie; Baron Larrey employs a gum elastic catheter, and M. Reynaud a bit of hemp, with injections, &c.

VI. *Injection.*—As the manner of performing this operation is familiar to every intelligent practitioner, we deem it unnecessary to translate the description of it by M. Malgaigne. Three accidents are enumerated as liable to attend this method: 1st. It sometimes happens during the operation that the canula slips out of the vaginal tunic, thus allowing the fluid to pass into the cellular substance of the scrotum. This accident, which can always be avoided by proper care on the part of the surgeon, is of a serious character, as it is liable to be succeeded by gangrene, unless the irritating fluid be immediately evacuated by free incisions. 2nd. The point of the trocar occasionally wounds one or more of the branches furnished to the scrotum by the internal or external pudic arteries, the epigastric, or finally the spermatic. In most cases, however, these branches are so small that their lesion is not of much consequence. 3rd. The instrument may enter the testicle. This accident is also rare, and usually produces no other inconvenience than a slight pain."

The operation is occasionally followed by violent inflammation and suppuration; and in one instance the writer of this article knew it to be succeeded by fatal tetanus. The patient, a stout, robust mechanic, about twenty-six years of age, whom he saw twice in consultation, was doing apparently very well during the first eight days after the operation; at this time, owing to exposure to cold, symptoms of the disease in question manifested themselves, and in less than twenty-four hours the man expired. Upon examination after death the vaginal tunic was found considerably thickened, and its cavity containing several ounces of sero-sanguinolent fluid with a small quantity of unhealthy looking pus. No adhesion had taken place between the opposite sides of the sac. A case of a similar kind is alluded to by Sir George Ballingall, in the second edition of his admirable "Outlines of Military Surgery," published at Edinburgh in 1838.

On the whole, our author seems to give injection a decided preference over the different methods we have described. When this operation, aided by compression, fails, I would suggest, he says, an attempt to obliterate the vaginal tunic by puncturing it with pins, as M. Bonnet does the sac in cases of hernia. He does not state that he has tried this plan, but thinks analogy warrants the belief that some good may be expected from it.

### 2. *Congenital Hydrocele.*

"The communication between the vaginal sac and that of the peritoneal forbids, for the most part, the employment of the procedures pointed out as applicable to ordinary hydrocele.

*Viguerie's Method.*—The patient lying on his back, the surgeon pushes the contents of the vaginal tunic into the abdomen by a sort of taxis, and applies upon the inguinal ring a herniary bandage or truss, which thus closes it up and prevents the fluid from re-descending.

*Method of Desault.*—This consists in puncturing and injecting the congenital hydrocele. Thus contrary to Viguerie, Desault forces the fluid as much as possible into the vaginal tunic, punctures the scrotum in the ordinary manner, and then, having ascertained that the intestine is fairly within the abdomen, he directs a trusty assistant to compress the external ring by means of a small pad, while he throws in the injection as in common hydrocele. To prevent any of the fluid afterwards from entering the peritoneal cavity, or the abdominal viscera from protruding, a truss should be worn until the cure is completed.

Of these two methods that of Viguerie is the more simple, as well as the less dangerous, and ought therefore to be preferred. Even the advanced age of the patient does not preclude the hope of a speedy obliteration of the ring. The methods of MM. Belmas and Gerdy, but above all that of M. Bonnet, for the radical cure of hernia, are likewise perfectly applicable here."

Such, to use a mercantile expression, is a fair sample of the work before us. Embracing the whole field of operative surgery, or, as the French term it, operative medicine, the accounts of the various mechanical procedures must necessarily be very brief, and, in many instances, imperfect. Hence one great objection to these manuals is, that they are calculated rather to mislead the student than to guide him to what is truly useful; in other words, their inevitable tendency is to create confusion and embarrassment as to what should be selected or what rejected. Many of the operations described by M. Malgaigne are no longer performed by the best surgeons, and they should therefore have been excluded from a work professing to be strictly elementary. Still, with all its blemishes, it is a very good production, which both the student and the practitioner may consult with advantage.

